# miR-6516-3p-mediated downregulation of the endogenous MMP-9 inhibitor RECK in mesangial cells might exacerbate lupus nephritis

**DOI:** 10.1186/s10020-025-01124-6

**Published:** 2025-03-05

**Authors:** Hiroyuki Tomita, Kunihiro Hayakawa, Keigo Ikeda, Hiroshi Tsushima, Marina Shinoura, Maki Fujishiro, Yuko Kataoka, Ken Yamaji, Kenji Takamori, Naoto Tamura, Iwao Sekigawa, Shinji Morimoto

**Affiliations:** 1 Institute for Environmental and Gender-Specific Medicine, Juntendo University Graduate School of Medicine, 2-1-1 Tomioka Urayasu-Shi, Chiba, 279-0021 Japan; 2 Department of Internal Medicine and Rheumatology, Juntendo University Urayasu Hospital, Chiba, Japan; 3 Department of Internal Medicine and Rheumatology, Juntendo University School of Medicine, Tokyo, Japan

**Keywords:** microRNA (miRNA), miR-6516-3p, Lupus nephritis, Systemic lupus erythematosus (SLE), Normal human glomerular mesangial cells (NHMC), RECK, MMP-9

## Abstract

**Background:**

MicroRNAs (miRNAs) regulate biological processes by inhibiting translation and causing mRNA degradation. In this study, we identified the miRNAs involved in the development and progression of lupus nephritis (LNs) and verified their roles.

**Methods:**

Total RNA, extracted from PBMCs collected from patients with LNs before and after treatment, was used for miRNA array analysis to identify miRNAs whose expression was significantly altered. The results of this analysis were confirmed using qRT-PCR. The identified miRNAs were transfected into normal human mesangial cells (NHMCs), human renal proximal tubule epithelial cells (RPTECs), human umbilical vein endothelial cells (HUVECs), and THP-1-derived macrophages (THP1-Mφ) to investigate their biological functions.

**Results:**

Three miRNAs were altered in PBMCs before and after treatment of LNs. Among these miRNAs, hsa-miR-6516-3p promoted TNF-α-induced expression of *MMP-9* in NHMCs. Moreover, hsa-miR-6516-3p downregulated the expression of RECK, an endogenous inhibitor of MMP-9. However, in NHMCs, endogenous hsa-miR-6516-3p was not present in functional amounts under inflammatory environment; therefore, we performed analysis using an experimental system considering extracellular influences of mesangial cells under LNs. The expression of hsa-miR-6516-3p was increased in HUVECs under inflammatory conditions and in activated macrophages.

**Conclusions:**

hsa-miR-6516-3p increases *MMP9* expression by suppressing RECK, and might, thereby, exacerbate LNs.

**Supplementary Information:**

The online version contains supplementary material available at 10.1186/s10020-025-01124-6.

## Introduction

Systemic lupus erythematosus (SLE) is an autoimmune disease that causes various symptoms and disorders in many organs, including the kidneys. More than 85% of SLE patients are female.

The highest incidence is often seen in the premenopausal years (ages 20–50 years) (Cooper et al. 1998). SLE develops due to abnormalities in innate and adaptive immune responses against genetic and environmental factors (Cooper et al. 1998). However, the detailed pathogenesis of the disease remains unknown, and the underlying cause for the diversity of symptoms is unclear.

Lupus nephritis (LNs), a major organ complication, is relatively common in more than 50% of SLE cases. LNs is a major risk factor for morbidity and mortality in patients with SLE. Approximately 10% of patients with LNs develop end-stage renal disease, and SLE patients with LNs die earlier than those without LNs (Almaani et al. 2017).

MicroRNAs (miRNAs) are noncoding RNAs that are approximately 22 nucleotides in length and are involved in the regulation of biological processes by inhibiting translation and degrading mRNAs (Hammond 2015). More than 2300 miRNAs have been identified in humans (Alles et al. 2019), which may regulate the expression of approximately 60% of human genes (Friedman et al. 2009). Recently, its clinical relevance has been suggested for various diseases, including malignancies (Calin and Croce 2006) (Rupaimoole and Slack 2017).

Altered miRNA levels are observed in most autoimmune diseases including rheumatoid arthritis and are recognized to influence autoimmunity through different mechanisms (Salvi et al. 2019; Zhang et al. 2023).

The association between SLE and miRNAs has also been widely reported. For example, anti-Su autoantibodies found in rheumatic diseases, including SLE (Satoh et al. 1994), recognize Argonaute2 (Ago2), an miRNA catalytic enzyme (Ikeda et al. 2006; Jakymiw et al. 2006). Aberrant expression of some miRNAs in CD4^+^ T cells in SLE, such as miR-21 and miR-148a, is associated with DNA hypomethylation, which is involved in SLE pathogenesis (Pan et al. 2010). Downregulation of miR-125b in T cells may contribute to SLE pathogenesis by regulating the expression of *ETS1* and *STAT3* (Luo et al. 2013). Thus, compelling data on miRNAs in SLE have accumulated, prompting suggestions that miRNAs may be potential biomarkers and promising therapeutic targets for SLE treatment (Zhang et al. 2020).

Previously, we reported that hsa-miR-4442 may be used as a biomarker for diagnosis of polymyositis and dermatomyositis and their disease activity (Hirai et al. 2018). Moreover, hsa-miR-766-3p identified in the plasma of patients with rheumatoid arthritis was reported to indirectly inhibit NF-κB activation. This mechanism partially contributed to the decreased expression of mineralocorticoid receptors by miR-766-3p (Hayakawa et al. 2019). However, these findings have not been led to further clinical studies.

In this study, we performed a comparative miRNA analysis using samples from pretreatment patients with active disease and post-treatment patients with inactive disease to identify LNs-specific miRNAs. We used peripheral blood mononuclear cells (PBMCs), which contain T and B cells involved in SLE pathogenesis, and in which miRNAs can be detected in a relatively stable form. Furthermore, we used cultured cells to examine the physiological functions of the miRNAs whose expression was found to be altered in LNs before and after SLE treatment. Our findings indicate that changes in miRNAs are involved in the pathogenesis of LNs.

## Materials and methods

### Patients and samples

Seventeen patients, one male and sixteen females, with LNs who were diagnosed and treated at the Department of Internal Medicine and Rheumatology, Juntendo Urayasu Hospital between June 2012 and June 2019 were included in this study. The mean age of the patients was 33 ± 15 years. All the patients met both the 1997 ACR classification criteria and the 2019 EULAR/ACR classification criteria. None of the patients had malignancies or acute or chronic infection. A random selection of eligible patients who met the criteria during the period examined in this study resulted in the male-to-female ratio (1:16) that was similar to the epidemiology data of SLE (Cooper et al. 1998). The SLE Disease Activity Index (SLEDAI) was scored based on clinical symptoms and laboratory tests. The mean pretreatment SLEDAI score was 18.5 ± 3.8 and the mean post-treatment SLEDAI score was 4.5 ± 4.0. The clinical characteristics of the patients are summarized in Table 1. The treatment of SLE was effective in all patients, as evidenced by a decrease in anti-double-stranded DNA antibodies, increase in CH50 (total activity of inactive C1-C9), decrease in urinary protein levels, and decrease in SLEDAI scores before and after treatment (Supplementary Fig. 1).

**Table 1:**
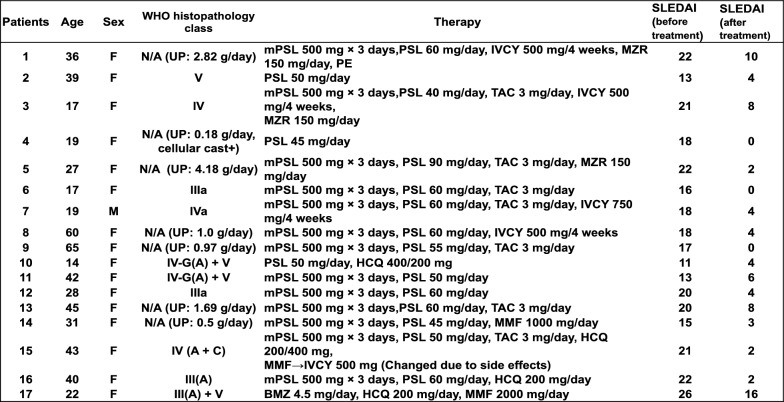
Clinicopathological characteristics of SLE patients with lupus nephritis

The histopathological classification was based on the tissue classification proposed by the International Society of Nephrology/Renal Pathology Society (ISN/RPS) in 2003 (Weening et al. 2004)

N/A: renal biopsy was not performed. UP urinary protein, mPSL methylprednisolone, PSL prednisolone, IVCY intravenous cyclophosphamide, MZR mizoribine, PE plasma exchange, TAC tacrolimus, HCQ hydroxychloroquine sulfate, MMF mycophenolate mofetil, SLEDAI SLE disease activity index

### miRNA array analysis

Total RNA was extracted from PBMCs before and after treatment of No.1–6 LNs patients (Table 1) using the miRNeasy mini kit (Qiagen, Hilden, Germany) and analyzed comprehensively using a miRNA array. The extracted RNA was labeled using a 3D-Gene™ miRNA labeling kit (TORAY, Tokyo, Japan), and the labeled targets were hybridized to a 3D-Gene Human miRNA 4-plex chip (V21_V1.0.0; TORAY). Hybridization images were scanned using a GenePix4400A device (Molecular Devices, Sunnyvale, CA, USA), and the miRNA levels were assessed based on the signal intensity, which was calculated as the median of the foreground signal minus the mean of the negative control signals + 2 standard deviations. In the absence of any definite internal control for miRNAs in PBMCs for standardization, the global normalization method was used for calculations.

### Cells

Normal human glomerular mesangial cells (NHMCs), human umbilical vein endothelial cells (HUVECs), and human renal proximal tubule epithelial cells (RPTECs) were obtained from Lonza (Basel, Switzerland). THP-1 cells were obtained from the ATCC (Manassas, VA, USA). NHMCs were maintained in Dulbecco’s modified Eagle medium (DMEM)/Ham’s F-12 (Wako, Osaka, Japan), supplemented with 10% fetal bovine serum (FBS). HUVECs were maintained in EGM™-2 BulletKit (Lonza) and RPTECs were maintained in REGM (Lonza) according to the manufacturer’s instructions. THP-1 was maintained in RPMI-1640 (Sigma-Aldrich Japan, Tokyo, Japan), supplemented with 10% FBS. THP1-derived macrophages (THP1-Mφ) were differentiated by adding phorbol 12-myristate 13-acetate (PMA) (50 ng/mL; Sigma-Aldrich Japan) to THP-1 for two overnight cultures and used for subsequent experiments.

### Transfection

miRNA mimics and S-TuDs (Synthetic Tough Decoy: miRNA inhibitor) were purchased from GeneDesign (Osaka, Japan). NHMCs were transfected with miRNA mimics (20 nM), S-TuDs (20 nM), or negative control (NC) miRNA/S-TuDs (20 nM) using Lipofectamine 3000 (Thermo Fisher Scientific, Waltham, MA, USA) according to the manufacturer’s instructions. After one overnight incubation, they were stimulated with TNF-α (10 ng/mL: R&D Systems, Minneapolis, MN, USA) for 24 h and were then subjected to quantitative reverse transcription polymerase chain reaction (qRT-PCR) analysis or western blotting, as described in a later section.

### Induction of miR-6516-3p by inflammatory stimuli

NHMCs and RPTECs were stimulated with TNF-α (10 ng/mL), IL-1β (10 ng/mL; R&D Systems), and IFN-α (50 ng/mL; Miltenyi Biotec, Bergisch Gladbach, Germany) for 24 h. HUVECs were stimulated with TNF-α (10 ng/mL), IL-1β (10 ng/mL), and IFN-α (50 ng/mL) for 6 h. THP1-Mφ were stimulated with lipopolysaccharide (LPS) (*Escherichia coli* 0111; B4, 1 μg/mL; Sigma-Aldrich Japan), TNF-α (10 ng/mL), IL-1β (10 ng/mL), or IFN-α (50 ng/mL) for 6 h. These cells were then subjected to qRT-PCR analysis.

### RNA extraction and qRT-PCR

Total RNA was extracted from the cells using the miRNeasy Mini Kit (Qiagen) according to the manufacturer’s instructions and reverse transcribed using the TaqMan MicroRNA Reverse Transcription Kit (Thermo Fisher Scientific).

Real-time PCR for miRNA measurement was performed using THUNDERBIRD Probe qPCR Mix (TOYOBO, Osaka, Japan) and specific primers (hsa-miR-4657, Assay ID: 462809_mat; hsa-miR-6894-5p, Assay ID: 466432_mat; hsa-miR-3607-3p, Assay ID: 463814_mat; hsa-miR-6516-3p, Assay ID: 467192_mat; hsa-miR-6126, Assay ID: 475618_mat; hsa-miR-769-5p, Assay ID: 001998; hsa-miR-150-5p, Assay ID: 000473; hsa-miR-4689, Assay ID: 463337_mat; U6 small nuclear RNA (U6 snRNA), Assay ID: 001973; Thermo Fisher Scientific) and QuantStudio 5 (Thermo Fisher Scientific) according to the manufacturer’s instructions. Cycle threshold (Ct) values were calculated using the ∆∆Ct method (Schmittgen and Livak 2008). The values for miRNAs extracted from each cell were normalized to that for U6 snRNA.

For measuring mRNA expression, total RNA was reverse-transcribed using the Prime-Script RT Reagent kit (Takara Bio, Shiga, Japan) according to the manufacturer’s instructions. Real-time PCR was performed using TB Green Premix Ex Taq (Takara Bio) and QuantStudio 5 according to the manufacturer’s instructions. The primer sequences for *IL1B*, *IL6*, *IL8*, *MMP9*, *RECK*, and *ACTB* are available on request.

### Western blotting

Cells were lysed in RIPA buffer (BioDynamics Laboratory, Tokyo, Japan) containing a protease inhibitor cocktail (Roche, Basel, Switzerland) and a phosphatase inhibitor cocktail (Thermo Fisher Scientific). The protein concentration was determined using a Micro BCA Protein Assay Kit (Thermo Fisher Scientific). The RECK (D8C7) rabbit monoclonal antibody was purchased from Cell Signaling Technology (Danvers, MA, USA). Anti-β-actin (AC-15) was purchased from Sigma-Aldrich. Horseradish peroxidase-conjugated anti-IgG secondary antibodies against rabbit IgG (Dako, Glostrup, Denmark) or mouse IgG (Cell Signaling Technology) were used with the Chemi-Lumi One substrate (Nacalai Tesque, Kyoto, Japan). Densitometric analysis was performed using the ImageJ software (Rasband, W.S., ImageJ, U.S. National Institutes of Health, Bethesda, MD, USA; http://rsb.info.nih.gov/ij/).

### Statistical analysis

Data from the miRNA arrays were analyzed using Microsoft Excel (Microsoft, Redmond, WA, USA). Statistical analyses of in vitro studies were performed using GraphPad Prism 9 software (GraphPad Software, La Jolla, CA, USA). Paired *t* test was used to compare the results for samples collected from patients before and after treatment. Statistical analysis of other in vitro studies was performed using the nonparametric Mann–Whitney *U* test to compare data from different groups. A *P* value < 0.05 was considered to indicate a statistically significant difference.

## Results

### hsa-miR-6516-3p significantly promoted TNF-α-induced expression of inflammatory genes, including MMP-9, in NHMCs

**Fig. 1:**
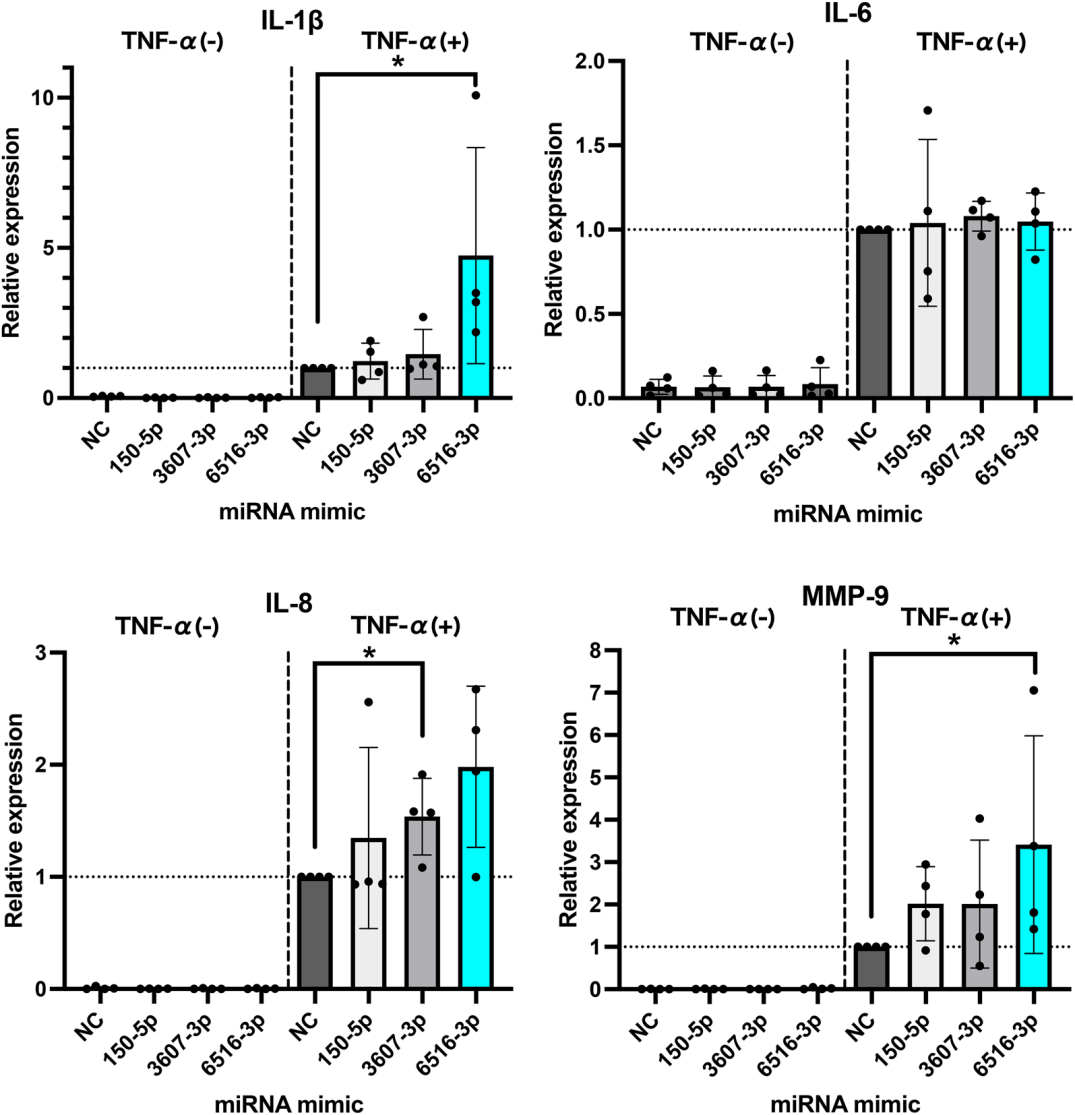
hsa-miR-6516-3p significantly promotes cytokine-induced expression of inflammatory genes. The effects of the miRNA mimics on the induction of inflammatory responses were analyzed using qRT-PCR. Normal human mesangial cells (NHMCs) were transfected with miRNA mimics miR-150-5p, miR-3607-3p, and miR-6516-3p and negative control (NC) miRNA. After two overnight incubations, cells were treated with TNF-α for 24 h and the expression of the indicated genes was evaluated using qRT-PCR. The dots represent the relative expression of each mRNA, and the column denotes the mean of data. Error bars indicate standard deviation. Each assay was performed four times. Asterisks indicate statistically significant differences (*P* < 0.05), as determined using the nonparametric Mann–Whitney *U* test

We performed a miRNA array analysis to identify miRNAs in PBMCs collected from LNs patients (No. 1–6) whose expression was changed before and after treatment. We selected miRNAs whose expression levels were significantly altered as determined using the mean fluorescence intensity and met the following criteria: (1) the variation in expression level was more than twice or less than half, (2) a significant difference was found using *t*-test, and (3) array results were stable (no deletions in detected samples). Nine miRNAs (hsa-miR-4657, 3653-3p, 6894-5p, 3607-3p, 6516-3p, 6126, 769-5p, 150-5p, and 4689) met the set criteria (Supplementary Table 1). However, miR-3653-3p was excluded from subsequent experiments because its primers were not available. Next, we performed qRT-PCR analysis on PBMCs (No. 1–17: Table 1) to verify the changes in miRNA expression. The expression of only three miRNAs, namely hsa-miR-150-5p, 3607-3p, and 6516-3p, was significantly altered in qRT-PCR analysis (Supplementary Fig. 2). Therefore, we investigated whether the expression of inflammatory genes was altered by miRNAs in mesangial cells under LNs conditions. NHMCs transfected with miRNA mimics were treated with TNF-α, and the expression of IL-1β, IL-6, IL-8, and MMP-9 mRNAs was analyzed using qRT-PCR. Only miR-6516-3p mimic significantly increased the expression of two inflammatory genes, *IL1B* and *MMP9*, in TNF-α stimulated NHMCs. *IL8* expression was significantly increased by miR-3607-3p, and an increasing trend was observed for miR-6516-3p. However, *IL-6* levels were not changed by any of the miRNA mimics (Fig. 1). Based on the abovementioned results, hsa-miR-6516-3p was thought to be a factor involved in LNs deterioration; therefore, we used this miRNA in subsequent analyses.

### hsa-miR-6516-3p suppressed RECK expression in NHMCs

**Fig. 2:**
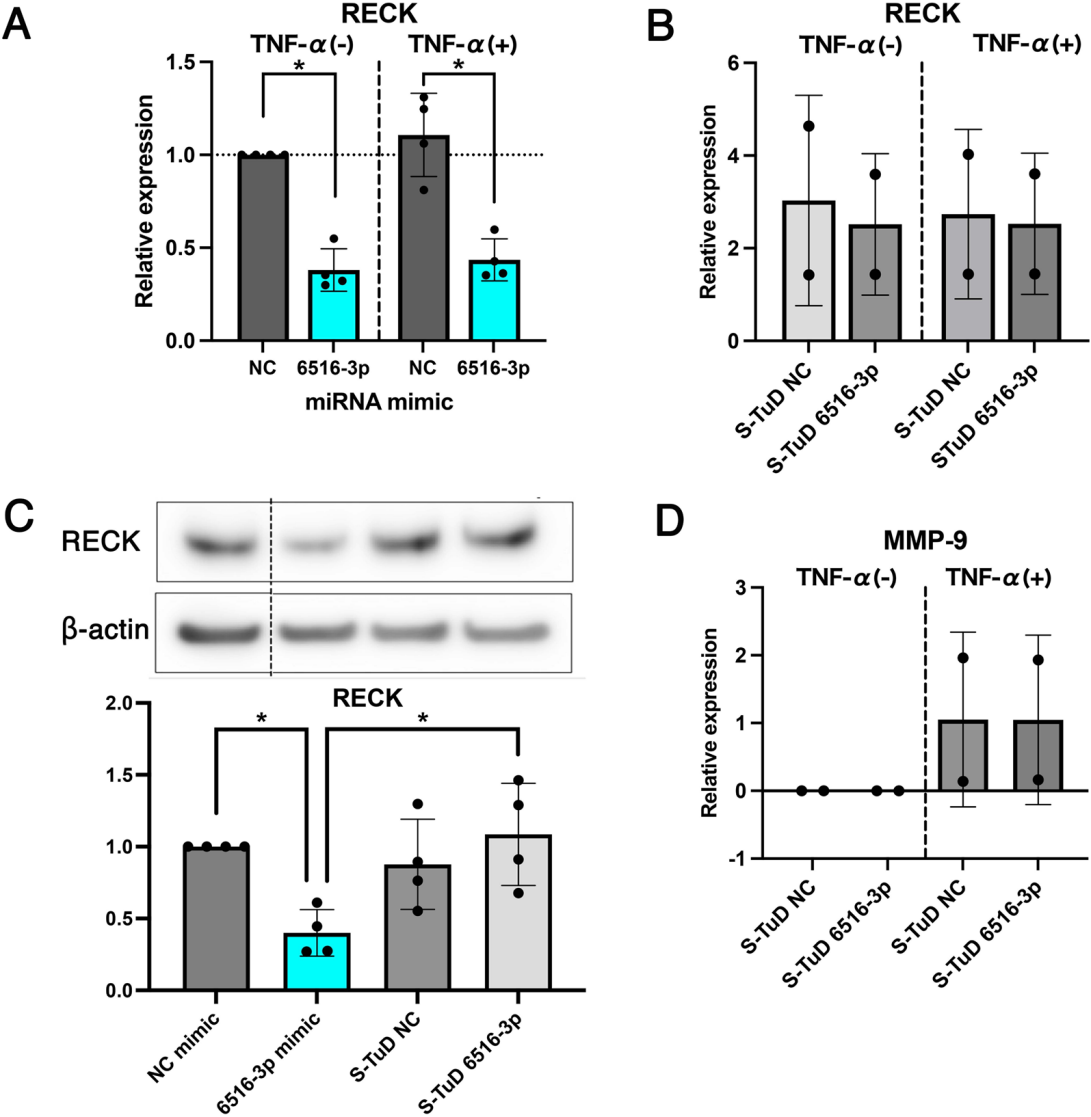
hsa-miR-6516-3p suppresses the expression of RECK. **A** NHMCs transfected with hsa-miR-6516-3p mimic were stimulated with TNF-α for 24 h and the expression of RECK mRNA was analyzed using qRT-PCR (*n* = 4). **B** RECK mRNA levels in hsa-miR-6516-3p S-TuD (hsa-miR-6516-3p inhibitor)-transfected NHMCs stimulated with TNF-α for 24 h were determined using qRT-PCR (*n* = 2). **C** The protein levels of RECK in hsa-miR-6516-3p mimic- or S-TuD-transfected NHMCs were determined using western blotting (WB) (*n* = 4). β-actin expression was used as a loading control. Upper panel shows representative protein bands of RECK and β-actin in WB. Lower panel shows the results of densitometric analysis of the bands in the upper panel. **D** The expression of MMP-9 mRNA in hsa-miR-6516-3p S-TuD-transfected NHMCs stimulated with TNF-α for 24 h analyzed using qRT-PCR (*n* = 2). The bars represent the means, and each dot represents an individual experiment. The error bars show standard deviation. A nonparametric Mann–Whitney *U* test was used to analyze the results. Asterisks indicate statistically significant differences (*P* < 0.05)

MMP-9 is particularly important in the progression of human glomerulonephritis (Urushihara et al. 2002). Reversion inducing cysteine rich protein with Kazal motifs (RECK) is an endogenous inhibitor of *MMP9* expression (Takagi et al. 2009). RECK levels are inversely correlated with the Systemic Lupus International Collaborating Clinics Damage Index in SLE (Hou and Zhang 2008). Therefore, we focused on the expression of RECK in NHMCs. First, we investigated the correlation between hsa-miR-6516-3p and *RECK* using TargetScan 8.0 (www.targetscan.org/vert_80/) (McGeary, Lin et al. 2019). Based on this analysis, hsa-miR-6516-3p was found to potentially bind to the 3′-untranslated region (UTR) of *RECK* (Supplementary Fig. 3). Therefore, we investigated the relationship between hsa-miR-6516-3p and *RECK* expression in NHMCs. Transfection of NHMCs with the hsa-miR-6516-3p mimic significantly reduced *RECK* expression, with or without TNF-α stimulation. Moreover, transfection of NHMCs with NC mimic also did not decrease *RECK* expression, even in the presence of TNF-α stimulation (Fig. 2A). Next, we examined whether the inhibition of endogenous hsa-miR-6516-3p in NHMCs promoted *RECK* expression. After transfection with hsa-miR-6516-3p inhibitor (miR-6516-3p S-TuD), NHMCs were stimulated with TNF-α and the expression of RECK mRNA was analyzed using qRT-PCR. However, there were no clear differences in *RECK* expression (Fig. 2B). We confirm these results using western blot analysis (Fig. 2C). Moreover, we determined the levels of MMP-9 mRNA in NHMCs transfected with miR-6516-3p S-TuD and treated with TNF-α. No significant changes in *MMP9* levels were observed (Fig. 2D). Taken together, the hsa-miR-6516-3p mimic decreased the expression of RECK in NHMCs, whereas the inhibition of endogenous hsa-miR-6516-3p did not change the expression of RECK. Therefore, the transfer of hsa-miR-6516-3p into NHMCs may contribute to the worsening of LNs by upregulating *MMP9* expression after suppressing RECK expression. Furthermore, endogenous hsa-miR-6516-3p might not be expressed in sufficient amounts to function in NHMCs.

### hsa-miR-6516-3p was upregulated in HUVECs and THP-1-derived macrophages by inflammatory stimuli

**Fig. 3:**
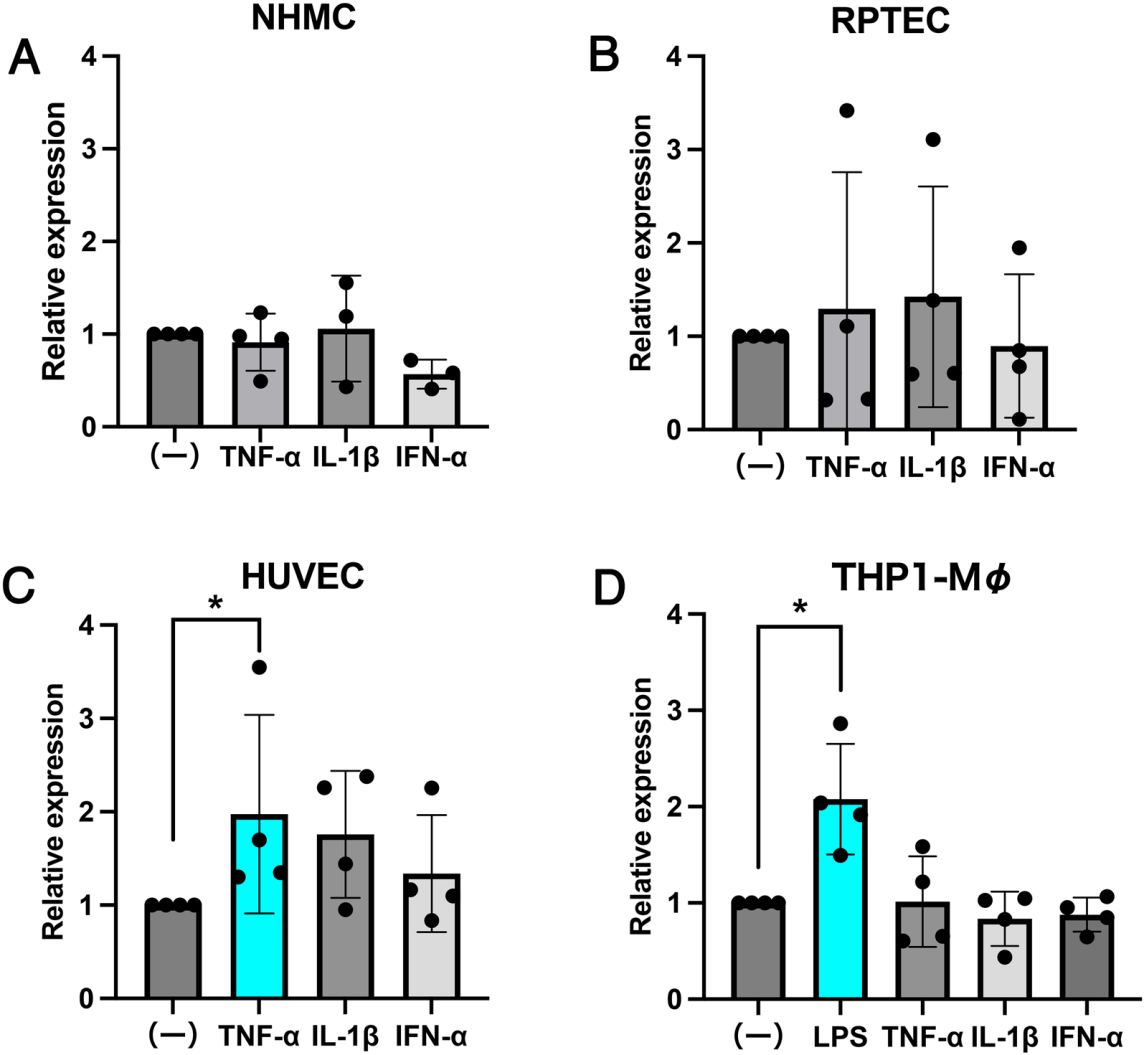
HUVEC- and THP-1-derived macrophages express hsa-miR-6516-3p under inflammatory stimuli. Using (**A**) NHMCs, (**B**) renal proximal tubule epithelial cells (RPTECs), (**C**) human umbilical vein endothelial cells (HUVECs), and (**D**) THP-1 derived macrophage (THP1-Mφ), the changes in the expression of hsa-miR-6516-3p during the induction of inflammation were validated via qRT-PCR analysis. **A** NMHCs and **B** RPTECs were stimulated with TNF-α, IL-1β, and IFN-α for 24 h. **C** HUVECs were stimulated with TNF-α, IL-1β, and IFN-α for 6 h. **D** THP1-Mφ were stimulated with LPS, TNF-α, IL-1β, and IFN-α for 6 h. The dots represent the relative expression of hsa-miR-6516-3p, and the column denotes the mean of data. Error bars indicate standard deviation. The assays were performed in quadruplicates. Asterisks indicate statistically significant differences (*P* < 0.05), as determined using the nonparametric Mann–Whitney *U* test

We investigated whether hsa-miR-6516-3p expression was affected in NHMCs under inflammatory conditions. We determined the hsa-miR-6516-3p levels in NHMCs treated with TNF-α, IL-1β, or IFN-α. The expression of hsa-miR-6516-3p in NHMCs did not increase after cytokine stimulation (Fig. 3A). Therefore, we investigated whether proximal cells influenced mesangial cells under LNs condition. We focused on RPTECs, glomerular endothelial cells, and macrophages. We verified whether the expression of hsa-miR-6516-3p in RPTECs changed during the induction of inflammation and whether hsa-miR-6516-3p was not significantly changed by cytokine stimuli (Fig. 3B). Next, we stimulated HUVECs, as a substitute for glomerular endothelial cells, as described for the previous experiment. Upon TNF-α stimulation, the expression of hsa-miR-6516-3p was significantly upregulated in HUVECs (Fig. 3C). Finally, we treated THP1-Mφ with LPS, TNF-α, IL-1β, or IFN-α and performed qRT-PCR analysis. LPS is a potent activator of immune cells, such as B cells, monocytes, and macrophages, and was added to activate macrophages in this study. LPS-activated macrophages exhibited a significant upregulation of hsa-miR-6516-3p expression (Fig. 3D).

Considering that miRNAs are secreted from cells, transmitted to other cells, and exhibit their functions (Kosaka et al. 2010; Squadrito et al. 2014), these data suggest that hsa-miR-6516-3p produced by glomerular endothelial cells and macrophages may affect mesangial cells under LNs. To test this possibility, we analyzed THP1-Mφ and HUVEC culture supernatants. Although hsa-miR-6516-3p was detected in the THP1-Mφ culture supernatant and its levels were high in the extracted exosomes, its effect on NHMCs could not be proven (data not shown).

In summary, we found that the expression of hsa-miR-6516-3p was significantly altered in PBMCs before and after treatment of patients with LNs. hsa-miR-6516-3p promotes an increase in *MMP9* levels in mesangial cells under inflammatory conditions by suppressing RECK expression, suggesting a possible association with LNs.

## Discussion

Recently, several miRNAs have been reported to be associated with the pathogenesis of LNs. The expression levels of miR-26a and miR-30b were decreased in the kidneys of patients with LNs and increasing the expression levels of these miRNAs in mesangial cells in vitro suppressed the expression of genes associated with mitosis and cell proliferation (Costa-Reis et al. 2015). miR-150 is upregulated in renal tissues, including the mesangial cells of patients with LNs, and promotes renal fibrosis by downregulating the suppressor of cytokine signal 1 (*SOCS1*) (Zhou et al. 2013). Moreover, elevated or decreased expression of specific miRNAs in PBMCs has been suggested to be related to disease activity in LNs. The expression of miR-98 in the PBMCs from patients with SLE correlates with LNs morbidity (Yuan et al. 2019), and the expression of miR-654 in the PBMCs from patients with LNs correlates negatively with urinary protein and serum creatinine levels (Tu et al. 2019). Using a miRNA array and qRT-PCR analysis, we identified three miRNAs, hsa-miR-150-5p, 3607-3p, and 6516-3p, whose expression was significantly altered before and after treatment in PBMCs from SLE patients with LNs (Supplementary Fig. 2). The main mechanism involved in the pathogenesis of LNs involves the induction of mesangial proliferative nephritis. We considered the possibility that these miRNAs may affect mesangial cells. Therefore, we performed transfection experiments using these three miRNA mimics in NHMCs. IL-1β, IL-6, and IL-8 are secreted by mesangial cells under inflammatory conditions, such as mesangial proliferative glomerulonephritis, including LNs (Brown et al. 1991; Liu et al. 2022). MMP-9, a protease that cleaves collagen IV, a major component of the glomerular basement membrane, plays an important role in the abnormal mesangial proliferative changes in human glomerulonephritis, including LNs (Urushihara et al. 2002). Therefore, we investigated whether the expression of inflammatory genes was altered by miRNAs in mesangial cells under LNs conditions. Only hsa-miR-6516-3p promoted TNF-α-stimulated expression of *MMP9* and *IL1B* significantly (Fig. 1). Urushihara et al*.* have shown that strong MMP-9 staining and gelatinolytic MMP-9 activity was increased in mesangial cells under inflammatory pathological conditions (Urushihara et al. 2002). In addition, as with *MMP9*, we analyzed the expression of *MMP2*, a group of gelatinases that degrade type IV collagen, which makes up the basement membrane, using qRT-PCR. In contrast to *MMP9*, *MMP2* expression was unchanged by miR-6516-3p (data not shown). Hence, hsa-miR-6516-3p, which increased the expression of *MMP9* and *IL1B* under inflammatory conditions may contribute to the exacerbation of LNs. Results of target gene analysis using miRNA database suggested that *RECK* is a target gene of hsa-miR-6516-3p (Supplementary Fig. 3). Indeed, the expression of both RECK was suppressed in NHMC by the hsa-miR-6516-3p mimic at both mRNA and protein levels (Fig. 2A and C). Takagi et al*.* showed that RECK decreased MMP-9 mRNA levels, but not the levels of other MMP mRNAs. Moreover, treatment with a RECK-specific siRNA increased MMP-9 mRNA expression in RECK-expressing cells. A promoter assay showed that the *MMP9* promoter activity was suppressed by RECK (Takagi et al. 2009). These findings and our data suggest that hsa-miR-6516-3p downregulates the expression of RECK, which in turn exacerbates LNs by increasing the *MMP9* expression.

Next, we investigated whether endogenous hsa-miR-6516-3p has physiological effects on NHMCs. We did not find any effect of the suppression of endogenous hsa-miR-6516-3p expression in NHMCs (Fig. 2B–D). Additionally, induction of inflammation did not alter the expression of endogenous hsa-miR-6516-3p (Fig. 3A). Previous studies have shown that miRNAs can be transferred to other cells (Kosaka et al. 2010; Squadrito et al. 2014). Therefore, we hypothesized that other cells may produce large amounts of hsa-miR-6516-3p, which may be transferred to mesangial cells in vivo. Our data showed that the levels of endogenous hsa-miR-6516-3p were significantly increased by inflammatory stimuli in both HUVECs and THP1-Mφ (Fig. 3C, D). We verified whether hsa-miR-6516-3p was released extracellularly from these cells and affected mesangial cells. hsa-miR-6516-3p was released from these cells into the culture supernatant, but in very small amounts, so the titer was not high enough to affect the mesangial cells (data not shown). This study had some limitations. Obtaining sufficient miR-6516-3p to affect mesangial cells in this experimental model would require a large number of cultured cells. Therefore, it is crucial to develop and verify an experimental model that can be used to demonstrate cell-to-cell communication to better reflect the in vivo environment. In particular, hsa-miR-6516-3p and mouse miR-6516-3p (mmu-miR-6516-3p, ID: MIMAT0027344) are highly homologous sequences, and in vitro and in vivo studies using a mouse model may be feasible.

Taken together, these results suggest that hsa-miR-6516-3p might be delivered to NHMCs from endothelial cells and macrophages around glomerular mesangial cells under LNs conditions, and that reducing the function of hsa-miR-6516-3p may be a therapeutic strategy for LNs.

## Conclusions

We discovered that hsa-miR-6516-3p contributes to an increase in the expression of *MMP9*, which is a factor that exacerbates nephritis, via the suppression of its endogenous inhibitor RECK. This mechanism may exacerbate LNs, and the inhibition of miR-6516-3p may be a new therapeutic tool for LNs.

## Supplementary Information

Additional file 1.

## Data Availability

No datasets were generated or analysed during the current study.

## Abbreviations

miRNA: MicroRNA
LNs: Lupus nephritis
PBMC: Peripheral blood mononuclear cells
qRT-PCR: Quantitative reverse transcription polymerase chain reaction
NHMC: Normal human glomerular mesangial cells
RPTEC: Human renal proximal tubule epithelial cells
HUVEC: Human umbilical vein endothelial cell
THP1-Mϕ: THP-1 derived macrophage
MMP: Matrix metalloproteinase
RECK: Reversion inducing cysteine rich protein with Kazal motifs
SLE: Systemic lupus erythematosus
SLEDAI: The SLE disease activity index
FBS: Fetal bovine serum
NC: Negative control
LPS: Lipopolysaccharide
Ct: Cycle threshold
