# Supplementary material for: miR-6516-3p-mediated downregulation of the endogenous MMP-9 inhibitor RECK in mesangial cells might exacerbate lupus nephritis
Source: Mol Med. 2025 Mar 5;31:84. doi: 10.1186/s10020-025-01124-6 (PMC11881388; doi:10.1186/s10020-025-01124-6)
Supplement: Supplementary file 1 — Additional file 1. [file 10020_2025_1124_MOESM1_ESM.docx]

**Supplementary Figure 1.** **Clinical data and disease activity before and after the treatment of SLE patients with lupus nephritis.** Blood and urinary protein levels were collected before and after the treatment of 17 patients with lupus nephritis. The dots on the left show the pretreatment values, and the dots on the right show the post-treatment values, and these are connected with a line for each patient. Asterisks indicate statistically significant differences (*P*< 0.05) determined using a paired *t*-test. SLEDAI: the SLE disease activity index.


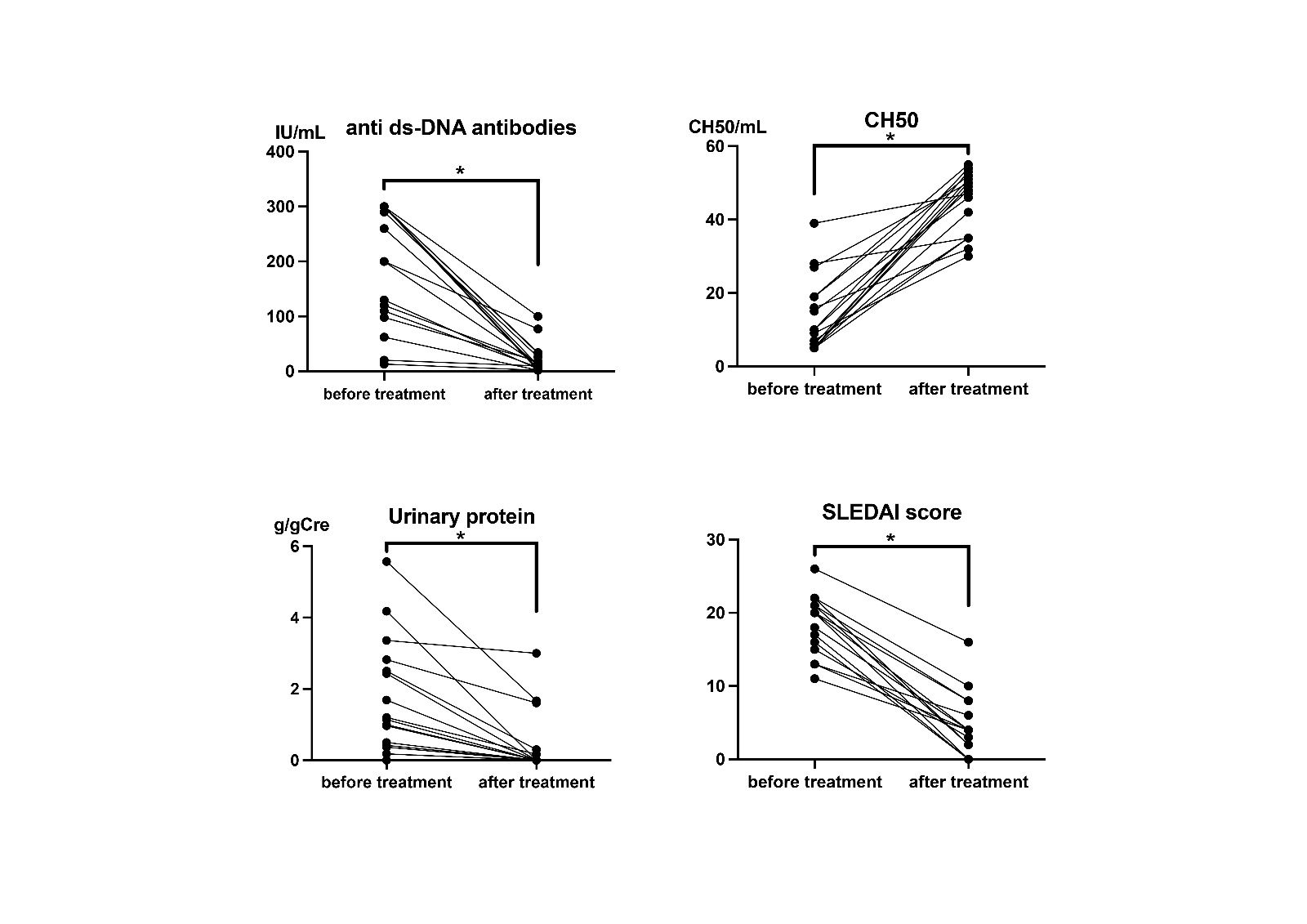


**Supplementary Figure 2. Validation of miRNA expression in PBMCs from patients with lupus nephritis.** qRT-PCR was performed to confirm differential expression of the indicated miRNAs in PBMCs before and after the treatment of patients with LNs (*n* = 17). The dots on the left show the relative expression before treatment and those on the right show the relative expression after treatment, and these are connected with a line for each patient. Asterisks indicate statistically significant differences (*P*< 0.05) determined using a paired *t*-test.


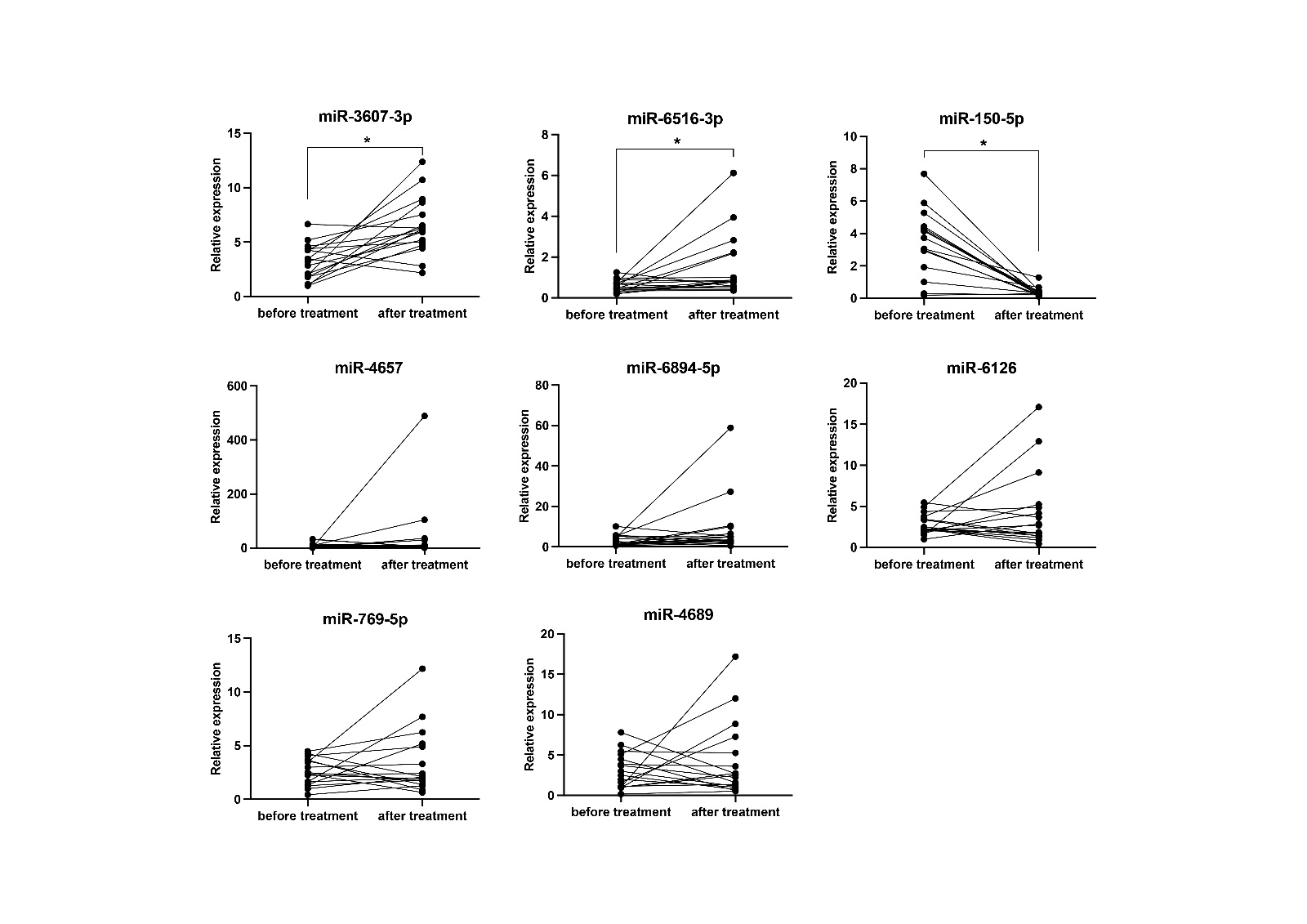


**Supplementary Figure 3. Reversion inducing cysteine rich protein with Kazal motifs (RECK) gene is a target of hsa-miR-6516-3p.** Predicted consequential pairing of the hsa-miR-6516-3p target regions in *RECK*. The upper and lower sequences are the hsa-miR-6516-3p sequences, and the middle sequence is the 3′-UTR of *RECK*. The hsa-miR-6516-3p sequence has the potential to bind complementarily to two sites in the 3′-UTR of *RECK*, at 1174–1180 and 1190–1197. On the upper and bottom sides, hsa-miR-6516-3p is shown to pair with the two target regions in the 3′-UTR of *RECK* (McGeary, Lin et al. 2019).


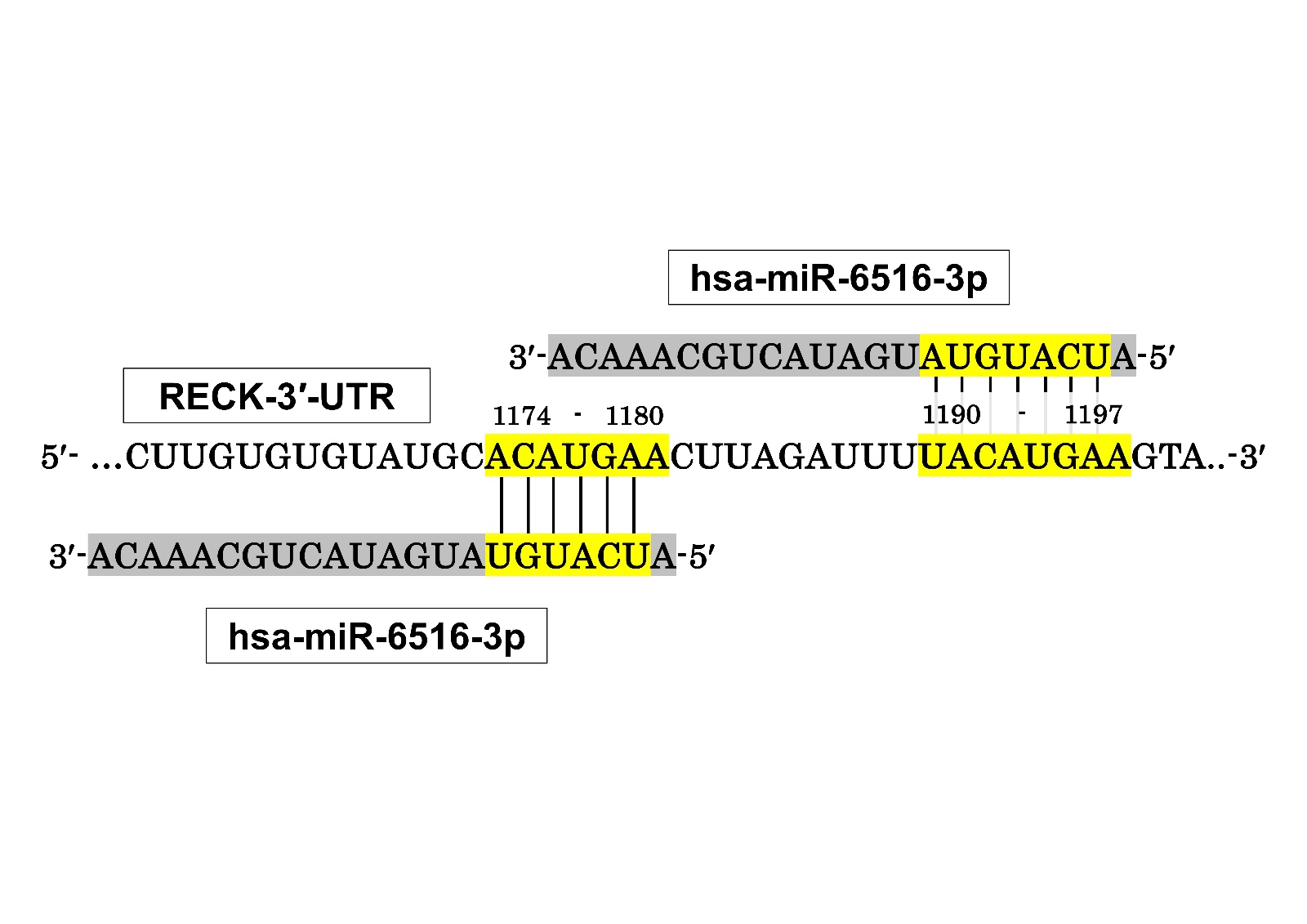


**Supplementary Table 1. miRNA array analysis of PBMCs from patients with lupus nephritis before and after treatment.**

| **miRNA Name** | **ID** | ***P value*** | ***Fold***  **(*before/after*)** |
| --- | --- | --- | --- |
| **hsa-miR-4657** | **MIMAT0019724** | **0.016** | **0.357** |
| **hsa-miR-3653-3p** | **MIMAT0018073** | **0.015** | **0.359** |
| **hsa-miR-6894-5p** | **MIMAT0027688** | **0.003** | **0.392** |
| **hsa-miR-3607-3p** | **MIMAT0017985** | **0.036** | **0.396** |
| **hsa-miR-6516-3p** | **MIMAT0030418** | **0.033** | **0.427** |
| **hsa-miR-6126** | **MIMAT0024599** | **0.044** | **0.429** |
| **hsa-miR-769-5p** | **MIMAT0003886** | **0.012** | **0.461** |
| **hsa-miR-150-5p** | **MIMAT0000451** | **0.028** | **0.467** |
| **hsa-miR-4689** | **MIMAT0019778** | **0.014** | **0.498** |
